# Clinical and Histopathologic Profile of Patients with Cutaneous Metastasis in a Tertiary Hospital in the Philippines

**DOI:** 10.3390/dermatopathology9040046

**Published:** 2022-12-08

**Authors:** Jolene Kristine Gatmaitan Dumlao, Eileen Liesl A. Cubillan, Juan Paolo David S. Villena

**Affiliations:** Department of Dermatology, University of the Philippines, Philippine General Hospital, Manila 1000, Philippines

**Keywords:** cutaneous metastasis, metastatic carcinoma, leukemia cutis

## Abstract

Introduction: Cutaneous metastases represent 2% of skin tumors, with an overall incidence of 5.3%. Although rare, clinical presentations of cutaneous metastasis vary and can be mistaken for benign and malignant skin conditions. Methodology: This was a descriptive, retrospective review of all patients diagnosed with cutaneous metastasis seen at the Department of Dermatology from January 2013 to December 2019. Clinical and histopathologic data from the patients were collated from medical records, and slides were retrieved for review. Results: A total of 115 patients were included and 122 slides reviewed. There were more female than males, the mean age was 52.3 ± 14.0 years of age. The most common primary cancer was the breast, and accordingly, the most common location was anterior chest. Among the 122 slides reviewed from 104 patients, the most common histologic type was adenocarcinoma (72.1%), and showed the infiltrative pattern (26.2%). Other histologic types seen were melanoma (13.1%), leukemic infiltrates (11.5%), squamous origin (2.5%), and epithelioid sarcoma (0.8%). Lymphovascular invasion and dermal sclerosis were observed. Immunohistochemical stains were performed in only 13.9% of the cases. There was a high concurrence of the clinical with the histopathologic diagnosis (95.6%). Conclusion: Although rare, patients with cutaneous metastasis may present in dermatology clinics. Knowledge of clinical features and low threshold for doing biopsies may prove useful for these patients. Similarly, dermatopathologists should be able to recognize histologic features of cutaneous metastasis morphologically. Histologic features may be subtle and may be reminiscent of benign inflammatory conditions, hence judicious use of immunohistochemical staining is recommended.

## 1. Introduction

Cutaneous metastasis is the infiltration of atypical cells in the dermis and subcutis, usually from a known primary malignancy. It heralds the progression, and recurrence of a previously treated malignancy. In some cases, it may be the initial presentation of an underlying internal malignancy [1,2,3]. Cutaneous metastases represent 2% of skin tumors, with an overall incidence of 5.3% [4]. Although relatively rare, it is an important condition encountered in dermatology and dermatopathology due to its varied clinical presentation, as well as its histologic presentation which may mimic primary cutaneous tumors [5]. A high index of clinical suspicion confirmed with histopathology is essential for early diagnosis and timely treatment [2].

Brownstein and Helwig first described the patterns of cutaneous metastasis in 1972 [6]. Cutaneous metastasis is diagnosed through clinical history, histomorphology of the lesion, and the comparison with the primary tumor if possible [1]. Several clues may point to a diagnosis of cutaneous metastasis including a primarily deep dermal infiltrate showing a “bottom-heavy” appearance, tumor cells arrayed as “nodules” and “strands”, grenz zone, and unremarkable epidermis [1,7,8]. However, the epidermis sometimes show ulceration and necrosis [1]. Cutaneous metastasis may also form glandular structures that resemble the primary malignancy [9]. Fernandez-Flores characterized the general morphological patterns for biopsies with cutaneous metastasis as nodular, infiltrative, diffuse, intravascular, top heavy, and bottom heavy [8]. Other histomorphologic features described in the literature are epidermotropism, necrosis, ulceration, lymphovascular invasion, and inflammation [1,3,5,8,9,10]. In some instances, immunohistochemistry (IHC) is performed. This is helpful in cases where the primary malignancy is unknown, or primary adnexal tumors are suspected.

In the Philippines, the literature search yielded only two published case reports of cutaneous metastasis in Filipinos, both of them have cutaneous metastasis originating from head and neck cancers. The first case report described a 70-year-old male with a 5-year history of untreated laryngeal squamous cell carcinoma. He presented with multiple nodules on the entire body three years after diagnosis of the primary malignancy. The patient was advised radiotherapy for which he refused. The patient expired due to cardiopulmonary complications few days after discharge against medical advice [11]. 

The other case is a 47-year-old male with nasopharyngeal carcinoma treated with radiotherapy for the primary carcinoma. Three years later, he presented with thickening of the left supraclavicular area with limitation of shoulder movement, initially assessed as radiation dermatitis. Biopsy of the lesion revealed nodular collection of neoplastic cells, some with mitosis, surrounded by moderately dense perivascular lymphocytic infiltrate, which was consistent with metastatic carcinoma. The patient underwent radiotherapy again with noted improvement of the skin lesions [12]. 

Studies on clinical and histopathologic characteristics of cutaneous metastasis are common in the Caucasian population. There are only few studies describing cutaneous metastasis in Asian populations, and usually the sample size of the cohort is small. This is the first study that describes these characteristics in the Filipino population and compare findings with those seen in the literature. Knowing the clinical and histologic characteristics of these patients contribute to updated information on cutaneous metastasis and may prove useful for future research and clinical practice on management.

## 2. Methodology

This was a descriptive, retrospective review of all patients diagnosed with cutaneous metastasis at the Department of Dermatology from January 2013 to December 2019. This study was reviewed and approved by the University of the Philippines Manila (UPM) Research Ethics Board (2019-425-01).

### 2.1. Data Collection

Clinical and histopathologic data from the patients were collated from the biopsy database of the department and medical records. The slides, including special stains if available, were retrieved. Only patients with biopsy-proven cutaneous metastasis from January 2013 to December 2019 were included. Cutaneous deposits from all malignancies, including internal organ cancers, hematologic malignancies (leukemia and lymphomas), and primary cutaneous malignancies such as melanoma, squamous cell carcinoma and Merkel cell carcinoma, were included. There were no set exclusion criteria for this study; however, missing charts and glass slides were documented. 

The search terms used were “cutaneous metastasis”, “metastatic carcinoma”, “metastatic adenocarcinoma”, “metastatic deposits”, “metastatic melanoma”, and “leukemia cutis”. Histologic sections were reviewed by the investigators. Data collected were entered into a standardized collection sheet with clinical and histologic information based on the related literature. Photos of the representative histopathologic slides were also collected.

### 2.2. Plan for Analysis

Quantitative data were summarized using means and standard deviation, while qualitative data were summarized as frequency and percentage. Histologic patterns and other pertinent histologic findings that cannot be summarized were described. 

## 3. Results

**Figure 1:**
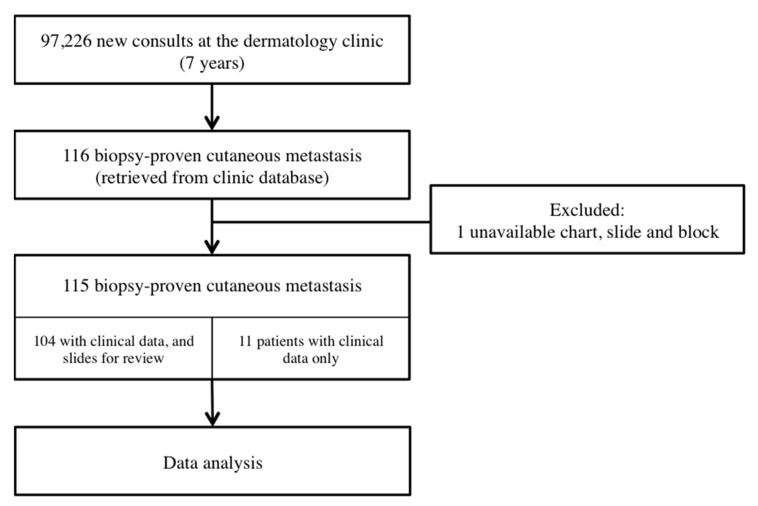
Study flowchart.

Among the 97,226 new cases seen at the dermatology clinic, there are 116 biopsy-proven cases with cutaneous metastasis. The incidence of cutaneous metastasis in this study is 0.12%. One case was excluded due to unavailable chart and slide. Among the 115 patients studied, 104 patients had available slides for review. A total of 122 slides were available from 104 patients (Figure 1).

**Figure 2:**
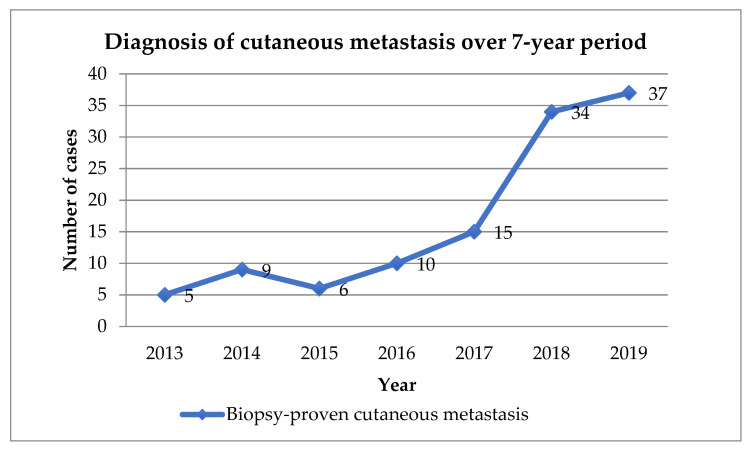
Diagnosis of cutaneous metastasis at the UP-PGH over the 7-year study period.

Over the years, there was an increasing trend of diagnosis of cutaneous metastasis (Figure 2).

### 3.1. Clinical Features

#### 3.1.1. Patient Characteristics and Location of Primary Malignancy

The mean age of the patients was 52.3 ± 14.0 years of age (range: 1–79 years old). There were more females (77.5%) compared with males (22.6%). The most common site of primary malignancy was the breast (61.7%). This was followed by hematologic malignancies (11.3%), melanoma (7.0%), malignancy of unknown origin (5.2%), and colorectal (3.5%). The most common malignancies seen in males were hematologic in origin, followed by unknown and colorectal, while in females the most common malignancies were located on the breast. The other primary malignancies seen are listed in Table 1. There were two patients with two malignancies: one was from a 63-year-old male initially treated for lung cancer, who then developed breast cancer 4 years after the resection; the other patient was a 64-year-old female who was treated for breast cancer, then developed cancer of the parotid 2 years after.

#### 3.1.2. Clinical Presentation of Skin Lesions

In this study, we found that majority of the patients presented with multiple (92.2%), skin-colored to erythematous nodules (50.4%), that did not present with secondary changes (79.1%). A combination of appearance of primary lesions were also observed. Of those with nodules, 34.4% were seen with plaques. In patients that presented with papules and plaques, 10.6% presented with induration, 6.4% with vesicles, and 2.1% with wheals. The majority of these lesions were asymptomatic (56.2%). Other associated symptoms seen were pruritus, infection, pain, and fluid accumulation such as edema and hydrocele (Table 2). In this study, it was found that the lesions had been present with a median time of 1 month (19.1%), ranging from 1 day to 2 years at the time of their consult.

#### 3.1.3. Sequence of Diagnosis between Primary Cancer and Cutaneous Metastases

Cutaneous metastases were the presenting sign of malignancy in three patients (2.6%). In 24 patients (20.1%) the sequence of diagnosis was synchronous, wherein the cutaneous metastases were diagnosed alongside the primary cancer. The most common sequence of diagnosis was metachronous, wherein the cutaneous metastases were diagnosed months or years later. This was observed in 63 patients (54.8%), in which the most common was 1–7 years post-therapy (71.4%). Five patients (4.3%) had late onset of cutaneous metastases, seen 10–19 years after the diagnosis and treatment of the primary cancer.

### 3.2. Location

**Figure 3:**
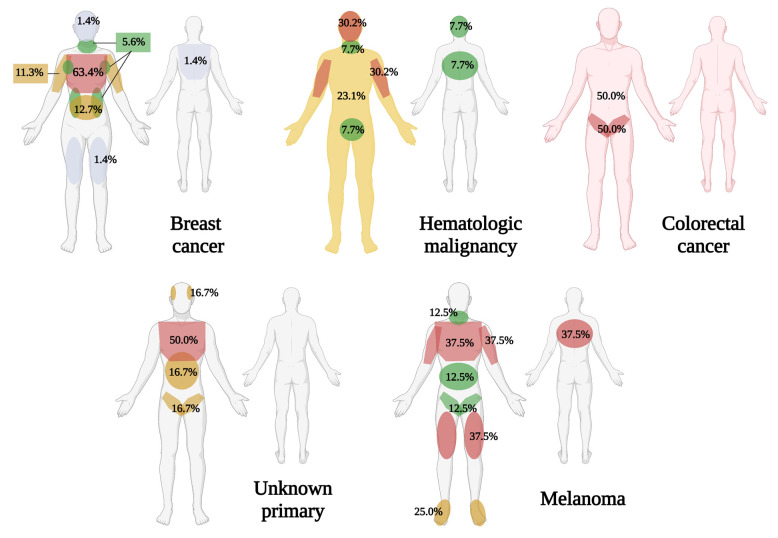
Location of cutaneous metastasis seen in the most common malignancies (Figure created with BioRender.com).

The most common location was subdivided per type of primary malignancy (Figure 3). In breast cancer, the most common site of metastasis was the anterior chest (63.4%), with 0.6% of these arising from the mastectomy scar. Other locations of metastasis were the abdomen (12.7%), arms (11.3%), neck (5.6%), axilla (5.6%), and flank (5.6%). Other sites noted were the back, scalp, face, and thigh.

The locations of metastasis seen in hematologic malignancies were found in the arms (30.2%), face (30.2%), generalized (23.1%), scalp, genital area, neck, extremities, and trunk. For melanoma, these were seen in the chest (37.5%), back (37.5%), arms (37.5%), legs (37.5%), foot (25.0%), neck (12.5%), abdomen (12.5%), and inguinal area (12.5%).

Metastasis from those with unknown primary malignancy were seen in the chest (50.0%), abdomen (16.7%), inguinal and scrotal area (16.7%), and the temporal area of the face (16.7%). While those with colorectal malignancies showed a generalized distribution (50.0%), and were in the inguinal area (50.0%).

### 3.3. Treatment

Overall, surgery was the most common treatment performed for the patients seen in 18.2% of the patients. Surgery with chemotherapy was performed in 15.7% of the cases. A combination of surgery, chemotherapy, and radiotherapy was performed in 13.9% of the patients. Chemotherapy alone was performed in 15.7% of the cases, most commonly performed in hematologic malignancies.

### 3.4. Histologic Features

Among the 122 slides reviewed from 104 patients, the most common histologic type was adenocarcinoma (72.1%). Other histologic types seen were melanoma (13.1%), leukemic infiltrates (11.5%), squamous (2.5%), and epithelioid sarcoma (0.8%).

**Figure 4:**
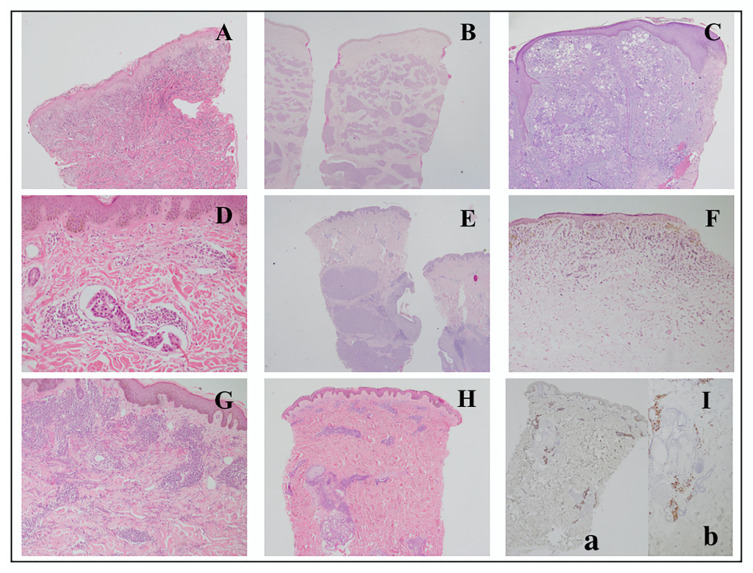
Representative photos of histologic patterns seen in this study. (**A**) Infiltrative pattern: Tumor cells insinuating in between collagen bundles in a breast cancer patient (4×); (**B**) Nodular pattern: tumor cells in nodules scattered throughout the dermis in an esophageal cancer patient (2×); (**C**) diffuse pattern: diffuse proliferation of atypical cells in a patient with unknown primary malignancy (4×); (**D**) intravascular pattern: atypical cells seen within a blood vessel with thin endothelial lining (20×). Patient has breast cancer; (**E**) bottom-heavy pattern: tumor cells that are nodules seen in a breast cancer patient (4×); (**F**) top-heavy pattern: strands of atypical cells seen mostly in the upper dermis (20×). Patient has breast cancer; (**G**) nodular and infiltrative pattern: tumor cells that are nodules and in strands seen in a leukemia cancer patient (20×); (**H**) superficial and deep perivascular pattern: mild superficial and deep perivascular infiltrates that are positive for myeloperoxidase stain (MPO) in a patient with leukemia (2×) (**I**) (**a**) MPO stain performed highlighting the atypical cells in case 101 (2×) (**b**) atypical cells also seen around periadnexal structures (10×).

The general patterns described in the study of Fernandez-Flores were observed, with the pattern of infiltrative being the most common (26.2%). Infiltrative pattern is shown in Figure 4A. Other histologic patterns described were nodular (Figure 4B), diffuse (Figure 4C), intravascular (Figure 4D), bottom-heavy (Figure 4E) and top-heavy (Figure 4F). However, a combination of these patterns was evident, and were described further in Table 3. Nodular and infiltrative patterns were distinct and were noted to be separate patterns (Figure 4G). Furthermore, one slide showed an inflammatory-like pattern (Figure 3H and Figure 4I(a,b)), superficial and deep perivascular and periadnexal pattern, which cannot be categorized in the pattern previously described in the literature. This pattern was observed in a case of hematologic malignancy.

A combination of nests, cords, strands, and sheets were seen as tumor patterns. The most common tumor pattern was nests, cords, and strands (45.1%). The other observed pattern was in sheets (10.1%), seen commonly in leukemic infiltrates and melanoma. Furthermore, glands and villi were seen in four cases, all observed in tumors of adenocarcinoma.

**Figure 5:**
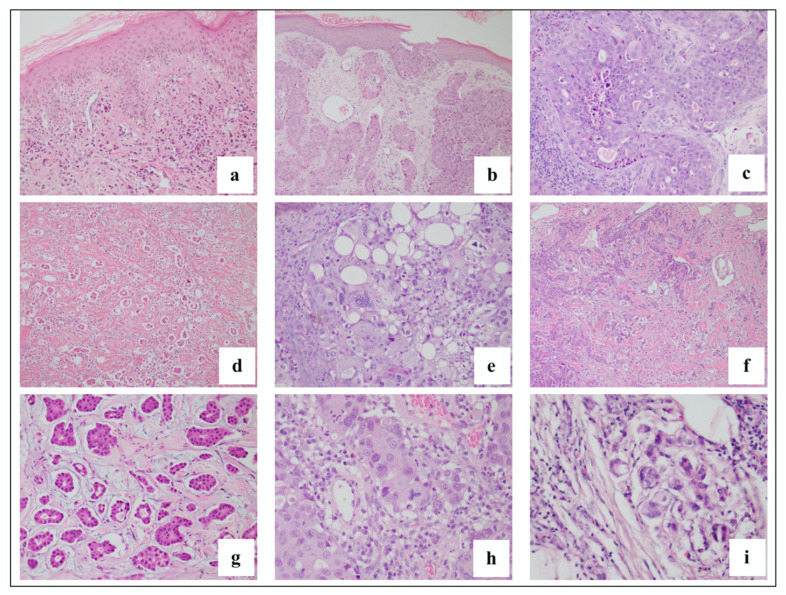
Histologic features seen in this study. (**a**) Epidermotropism seen in case 1302, from breast adenocarcinoma (20×). (**b**) Necrosis and ulceration in a case of breast adenocarcinoma, 1818 (40×). (**c**) Tumor necrosis in a case of breast adenocarcinoma, case 1708 (40×). (**d**) Atypical cells interspersed between collagen bundles, and within vascular spaces resembling florets, seen in the entire dermis (20×). (**e**) Atypical pleomorphic cells with high mitotic index seen in case 1701, from a case with unknown primary malignancy (40×). (**f**) Hints of mucin and sclerotic stroma seen in a case of colorectal adenocarcinoma, case 1807 (20×). (**g**) Wisps of mucin surrounding tumor cells in a case of breast adenocarcinoma, case 1818 (40×). (**h**) Mixed cell infiltrate showing lymphocytes, neutrophils, eosinophils and plasma cells, in a case with unkown primary malignancy, case 1828 (40×). (**i**) Signet ring cells seen in a a case of breast adenocarcinoma, case 1706 (40×).

Epidermotropism was present in 24.6% of the slides (Figure 5a). Of these, 66.7% were from adenocarcinoma, 23.3% were melanoma, 6.7% were leukemic infiltrates, and 3.3% from sarcoma. Ulceration was present in 17.2% (Figure 5b). Tumor and epidermal necrosis were seen in 11.5% (Figure 5c) and 10.7%, respectively. There were 3.3% cases which showed both tumor and epidermal necrosis.

Lymphovascular invasion was present in 90.2% of the slides reviewed (Figure 5d), if hematologic malignancies were excluded, lymphovascular invasion was present in 90.7% of the cases. Mitotic figures were identified in 69.7% of the specimens (Figure 5e). Of these, 67.1% displayed low mitotic activity, 22.4% moderate, and 10.6% high mitotic activity. A total of 45.9% specimens had neural invasion.

There were dermal changes observed in 62.3% of the specimens. Of these changes, the most common was sclerosis (53.4%), followed by sclerosis with mucin (Figure 5f) around the tumor (14.5%), fibrosis around the tumor proliferation (13.2%), mucinous stroma (9.2%) (Figure 5g). Keloidal stroma was observed in one case, and the remaining changes seen were vascular in nature (increase in vessels and congestion in vessels).

In assessing the inflammation, leukemic infiltrates were not included because immunohistochemical stains were needed to assess whether the inflammatory cells, particularly lymphocytes, were part of the malignant proliferation. Of the 108 slides reviewed, 74.1% showed sparse inflammatory infiltrate and 20.4% were moderately dense, predominantly lymphocytes. Eosinophils were observed in 29.5% cases, plasma cells in 4.8%, and mast cells were seen in 1.9% of those with inflammatory infiltrates (Figure 5h). Interestingly, neutrophils and leukocytoclasia were seen in 6.7% of the cases. Signet ring cells were also observed (Figure 5i).

Immunohistochemical stains were performed in only 13.9% of the cases. Forty-seven percent were performed to confirm adenocarcinoma, 23.5% to confirm leukemia, 23.5% for melanoma, and 5.9% for squamous in origin. Table 4 summarized the stains performed.

### 3.5. Concurrence of Diagnosis

There was a high concurrence of the clinical diagnosis with the histopathologic diagnosis at 95.6%. Cutaneous metastases were the primary consideration in these cases, if not part of the differential diagnosis provided by the clinicians (Table 5).

## 4. Discussions

Cutaneous metastasis develops as a result of genetic factors, epigenetics, and host response [7]. It travels from the primary to distant sites through hematogenous and lymphatic spread, directly connecting tissue invasion, and iatrogenic implantation [2,13]. In order to metastasize, tumor cells should be able detach from the primary site, invade extracellular basement membrane, enter the circulation, evade host defense mechanisms, incite vessel proliferation, and respond to paracrine growth factors. The metastatic cascade is assisted by classes of molecules associated with cell invasion, cell adhesion and growth factors. All of these processes are crucial for metastasis to occur [2,7,8].

There are two regarded hypotheses in the mechanism of metastasis, the clonal expansion hypothesis and the rare variant model. The clonal expansion hypothesis suggests how genetic predisposition to activation of specific oncogenes and loss of tumor suppressor genes, play a huge role in giving advantageous potential to tumor cells. This advantage may come in the form of growth autonomy, angiogenesis, resistance to anti-growth signals, evasion of apoptosis and capability to invade and destroy tissue, and to metastasize. These changes allow tumor cells to proliferate through clonal expansion 7]. The rare variant model, on the other hand, considers that there is a minority of aggressive tumor cells that “pre-exist” in a primary tumor that has the capacity to form metastasis; hence, the small population of those with primary cancers having cutaneous metastasis [7].

A summary of published retrospective studies on cutaneous metastasis from different countries is provided (Table 6) [1,3,5,8,9,10,13,14,15,16,17]. In some of these studies, metastatic melanoma and lymphomas were included [1,3,8,9,10,13,16].

This study reviewed 115 patients with cutaneous metastasis over a seven-year period, which showed a large cohort of patients in a short period of time compared with prior studies. The study site, a tertiary government hospital in the country, caters to thousands of Filipino patients from across the country, and is also considered an end-referral hospital for cancer cases that need to be seen by specialists including medical oncologists and radiation oncologists. This hospital is also an academic teaching institution that trains physicians and specialists in the country.

This study showed an overall incidence of 0.12% in patients seen in dermatology, with incidence of cutaneous metastasis calculated based on the total number of new patients seen in the dermatology clinic. Previous studies have calculated based on autopsies and previous case series on cancer patients [4]. The incidence is much lower compared with prior studies; however, it should be noted that this value is based on patients seen in the dermatology clinics and not the incidence of metastasis seen in cancer patients. Patients with cutaneous metastasis consult with dermatologists for the skin lesions, and although it is rare, the diagnosis or the consideration of it, should not be missed. Furthermore, there was an increasing trend of diagnosis by the dermatologists in the institution, which may represent an increase in vigilance, as well as improvement of clinical acumen of clinicians in the training hospital. 

The mean age of the patients as well as the sex distribution seen in this study was similar to the available literature on cutaneous metastasis, ranging from 55.2 to 67.9 years of age [1,3,5,9,10,13,14,15,17]. Since this study included leukemia cutis, patients with hematologic malignancies tend to be younger (range: 1–52 years of age), with the youngest patient in this study being 1 year of age.

The most common primary malignancy to metastasize in females was from the breast which is also consistent in the literature [3,5,9,10,14,15,16]. On the other hand, there are differences in the most common primary malignancy and the location in males. The most common primary malignancies in the literature are carcinomas from the lungs [1,5,6,14], gastric and colorectal [1,13,17], and if included in the study, melanoma [1,10] and Non-Hodgkins lymphoma [3]. Among Asians in Singapore, unknown primary malignancy was the most common in males [15]. The most common source of cutaneous metastasis seen in males in this study was from hematologic malignancies. Melanoma was the second most common in males in this study, similar to Kaplan [10] and Saeed [1]. However, the majority of the studies seen on cutaneous metastasis excluded hematologic malignancies because of its inherent capability to circulate in the body, and also melanoma, due to its proximity to the skin. Hence, if both conditions are excluded from analysis, the data would approximate that of Gan and colleagues [15] from Singapore, wherein the malignancy was from an unknown primary malignancy.

The most common clinical presentation was asymptomatic multiple nodules, which also approximates those in the previous literature [1,8,10,13,15]. The most common presentations are solitary or multiple asymptomatic nodules, although cutaneous metastasis may present as inflammatory lesions (plaques) with epidermal changes such as necrosis, or telangiectatic skin lesions because of vascular changes. Other less common forms are alopecia, morphea-like (en cuirasse), and cellulitis-like morphology [7]. Furthermore, other lesions may resemble benign skin conditions such as pyogenic granuloma, granular cell tumor, epidermal inclusion cysts, picker’s nodule, herpes zoster, hemangiomas, and adnexal tumors, among others [5,7,13,15]. Hence, a skin biopsy of the lesion is valuable in differentiating cutaneous metastasis from other skin conditions.

In terms of location, because the most common primary malignancy to metastasize was from the breast, it was fitting that the chest, trunk, and abdomen were the most common site of predilection [3,5,9,10,13,14,17]. Other common areas are the head and neck, particularly the scalp. In this study, we also showed differences in the predilection sites of metastasis based on the type of malignancy. In a large study that looked into the behavior of T cells (regulatory T cells, CD4 effector T cells, and CD8 T cells) in cutaneous metastasis through flow cytometry, Schulman and colleagues were able to identify that areas with high T_reg_ density and CD4:CD8 ratio were most permissive to tumor growth [18]. The predilection areas of cutaneous metastasis which showed high heat maps were those contiguous to existing primary malignancy (i.e., chest in breast cancer, groin area in colon cancer), and those with extensions from underlying lymph node basins (i.e., inguinal area in melanoma) [18]. In their study, they were able to identify that the head and neck area also exhibits high immunologic factors. There is a preferential localization of T_reg_ to hair follicles, hence the high percentage of spread to the scalp [18]. In particular, patient 106 initially presented with an asymptomatic nodule on the scalp without prior history of malignancy, where histology showed clear cells that pointed to cutaneous metastasis that may be renal in origin.

The particular instance mentioned above showed how the cutaneous metastasis may precede the diagnosis of the primary malignancy. Cutaneous metastasis may occur along with the symptoms of the primary malignancy, or may also signify recurrence of a previously treated disease. However, in some cases, it may be the presenting sign of an underlying malignancy. In 50–81.8% of the cases, cutaneous metastasis are suspected by the primary clinician [1,5,13]. In terms of the sequence of diagnosis of primary malignancy and cutaneous metastasis, the majority of patients (54.8%) had a metachronous presentation, wherein the cutaneous metastasis were diagnosed months or years after therapy. Of these, 71.4% presented within 1–7 years after diagnosis. Cutaneous metastasis is usually suspected in patients with a known primary malignancy and are consulting for new-onset lesions [13] in up to 88% of the patients in the study of Sariya et al. [5]. Lung cancer and gastrointestinal malignancies usually present with early cutaneous metastasis, as opposed to breast cancer, where the cutaneous metastasis may show several years after [13]. However, some authors have described that cutaneous metastasis was the first sign of malignancy seen in their studies [5,13,14,17], seen in up to 54% of the cases [15]. In this study, we only saw three patients (2.6%), with the cutaneous metastasis seen as the first presentation. A small percentage (4.3%) also presented late, with one patient presenting with skin lesions 19 years after the primary cancer. Although seen only in a small percentage of this population, clinicians as well as dermatopathologists should be careful because of the possibility of cutaneous metastasis. The time to appearance of cutaneous metastasis from the initial diagnosis of the internal cancer ranged from less than one month to 28.2 years [1,3,9,13,14,15]. In this study, the lesions were present with a median time of 1 month (19.1%), ranging from 1 day to 2 years at the time of their consult.

Another interesting finding is that the clinicians in this study had the highest concurrence of the clinical diagnosis with the histopathologic diagnosis, seen at 95.6% of the cases, with other literature having the clinical suspicion of metastasis reported ranging from 55% [5], 72% [15], 75.3% [1], and 81.8% [8]. Similar to the study of Saeed, lesions were clinically suspicious for both benign and malignant entities [1].

Hussein described the following features that could differentiate cutaneous metastasis from primary skin malignancies: tumor cells in lymphatic and blood vessels, tumor cells in the deep dermis and subcutaneous fat, and neoplastic cells in between collagen bundles [7]. Basic histopathologic patterns of cutaneous metastasis were described as “nodular” and “in strands” [1]. Alcaraz et al. described four major patterns: nodular, infiltrative (interstitial), diffuse, and intravascular [2]. Fernandez-Flores further described the bottom-heavy and top-heavy pattern [8]. This study showed that cutaneous metastasis indeed demonstrated the occurrence of the six patterns described, as well as the combination of these patterns. The most common pattern seen in this study was infiltrative, followed by intravascular, and nodular. Tumor cells were most commonly arrayed in nests, cords, and strands. The infiltrative pattern shows how the tumor cells insinuate between collagen bundles, which may mimic benign inflammatory conditions such as interstitial granuloma annulare [19].

Previously, hematologic neoplasms were described to show a “bottom-heavy” pattern [8]. However, in this study we saw that these neoplasms may also exhibit infiltrative, nodular, and diffuse patterns, similar to cutaneous metastasis from other malignancies. Of note, one case showed a superficial and deep perivascular pattern, which needed the use of immunohistochemical (IHC) staining to prove the diagnosis. Hence, a clinical history as well as appropriate use of immunohistochemical stains proved valuable in the diagnosis of these cases.

There were histologic features that differ from previously reported studies. Lymphovascular invasion was high in this study, seen in 90.2% of the slides reviewed, and 90.3% if we disregard hematologic malignancies. This is high compared with the 25% previously reported [1,5]. Epidermotropism was seen in 24.6% of the slides, which was higher compared with the rare occurrence in Fernandez-Flores (9 out of 78 biopsies) [8]. 

Ulceration and necrosis was low in this study, as well as sparse infiltrates seen in the study similar to the findings of Fernandez-Flores [8]. Intratumoral neutrophils within glands, previously believed to be suggestive of colon carcinoma [1,8], were also observed in the two cases of colorectal carcinoma in this study. However, neutrophils were also observed in other cases of adenocarcinoma seen in this study. Eosinophils were seen in 29.5% of the cases. Eosinophils were regarded to be anti-tumorigenic in several cancers such as gastric, colorectal, oral, nasopharyngeal, and breast cancer, but were considered to be indicators of poor prognosis in other cancers such as ovarian, cervical, lung cancers, and some lymphomas [20]. 

Sclerotic stroma and keloidal collagen was seen in this study, particularly in the infiltrative pattern, similar to the findings of Panjwani et al. Careful search of other features point to cutaneous metastasis, along with the help of IHCs, since this finding may also be seen in dermatofibroma [19].

Immunohistochemical staining was performed in 13.9% of the cases in this series. They were useful in cases where the morphologic features were not distinguishable. IHC was helpful particularly in patient 81 with two previous primary malignancies, breast, and lung cancer. Clinically, the lesion looked like angiosarcoma; however, using the panel of IHCs ER was positive, which pointed to a diagnosis of cutaneous metastasis from breast primary. ER and PR receptors have been helpful in diagnosing cutaneous metastasis from the breast [1]. 

The usual panel performed are the cytokeratins: CK7, CK20 (to determine glandular epithelium), SOX10 (for melanoma, neural, and myoepithelial tumors), and p63 (negative staining is helpful to rule out primary cutaneous carcinoma, and positive staining rules in squamous cell carcinoma and other primary adnexal carcinomas) [21]. CK5/6 and podoplanin may be helpful in distinguishing primary adnexal tumors from cutaneous metastasis [1,17]. Other recommendations are CD45 (lymphoid malignancies), AE1/A3 pancytokeratin (cytokeratin for most carcinomas), S100 (melanoma), CD34 (vascular neoplasms), epithelial markers (EMA and CEA), chromogranin (neuroendocrine tumors), prostate-specific antigen (prostate cancer), thyroid transcription factor (for lung cancer), WT1 (ovarian carcinoma), CDX2 (intestinal carcinomas), and Hep Par1 (hepatocellular carcinoma) [7].

However, in cases where immunoperoxidase stains are not helpful due to identical phenotypes of cells, pathologists and dermatopathologists rely on clinicopathologic and histomorphologic information [1]. Immunohistochemistry (IHC) should be used judiciously, as there are no antibodies that are pathognomonic of a specific diagnosis, especially if used on its own. IHC should be performed on the appropriate morphologic context [8].

Management of cutaneous metastasis is the treatment of the primary malignancy, hence referrals to the appropriate specialties such as medical oncology and radiation oncology are indicated. In the advent of advanced treatment of cancers, there is better survival for patients, and thus they are prone to develop sequelae of advanced disease such as cutaneous metastasis [22]. These patients experience poor quality of life due to the risk of infection, bleeding, disfigurement, and pain. Hence, skin-directed therapies such as intralesional and topical therapy of antineoplastic agent, photodynamic therapy, electrochemotherapy, and radiotherapy for cutaneous metastasis have been explored [22].

The median survival of patients in one study is 10 months, with melanoma having better prognosis than breast cancer and others with unspecified malignancy [10]. In the study of Hu et al., they noted that there are better overall survival rates for patients with breast cancer and skin involvement only, compared with those with breast cancer, visceral and skin involvement, and non-breast cancer. The prognosis of the patient largely depends on the aggressive behavior of a particular cancer, rather than just the appearance of the skin metastasis [13].

Due to the retrospective nature of the study, there were several limitations seen. Treatment performed for cutaneous metastasis after its diagnosis, as well as the outcome of the patients were not adequately identified due to incomplete data. It is also important to note that some of the slides were not optimal due to the time in storage, hence, some histologic features such as the appearance of mucin may not be fully appreciated. A prospective study may be performed to assess the incidence of cutaneous metastasis, especially for the most common primary tumor seen such as in the patients in the breast cancer clinic.

## 5. Conclusions

Although rare, patients with cutaneous metastasis may present in dermatology clinics. Full clinical and physical examination should be performed in patients with prior history of malignancy. The head and neck region, particularly the scalp should not be overlooked. Knowledge of clinical features and a low threshold for doing biopsies may prove useful for these patients. Similarly, dermatopathologists should be able to recognize histologic features of cutaneous metastasis morphologically. The infiltrative pattern and lymphovascular invasion are features that should be carefully searched for. Some histologic features are subtle and may be reminiscent of benign inflammatory conditions, hence judicious use of immunohistochemical staining is recommended.

## Figures and Tables

**Table 1:** Site of location of primary cancer and sex distribution.

Site	Total (*N* = 115)	Male (*n* = 26)	Female (*n* = 89)
	*N* (%)	*n* (%)	*n* (%)
Breast	71 (61.7)	0	71 (79.8)
Hematologic	13 (11.3)	7 (26.9)	6 (6.7)
Skin (melanoma)	8 (7.0)	4 (15.4)	4 (4.5)
Unknown	6 (5.2)	4 (15.4)	2 (2.2)
Colorectal	4 (3.5)	2 (7.7)	2 (2.2)
Thyroid	2 (1.7)	1 (3.8)	1 (1.1)
Bladder	1 (0.9)	1 (3.8)	0
Breast and Lung	1 (0.9)	1 (3.8)	0
Breast and Parotid	1 (0.9)	0	1 (1.1)
Cervix	1 (0.9)	0	1 (1.1)
Esophagus	1 (0.9)	1 (3.8)	0
Lung	1 (0.9)	1 (3.8)	0
Nasopharyngeal	1 (0.9)	1 (3.8)	0
Pancreas	1 (0.9)	1 (3.8)	0
Renal	1 (0.9)	1 (3.8)	0
Soft tissue	1 (0.9)	1 (3.8)	0
Vulva	1 (0.9)	0	1 (1.1)

**Table 2:** Clinical presentation of the skin lesions of patients diagnosed with cutaneous metastasis (*N* = 115).

	*N* (%)
**Symptoms associated with skin lesions at presentation**	
None	65 (56.5)
Pruritus	16 (13.9)
Infection (yellowish crusts, draining abscess)	14 (12.2)
Pain, tenderness	12 (10.4)
Lymphedema	5 (4.3)
Not known	2 (1.7)
Hydrocele	1 (0.9)
**Clinical presentation of lesions**	
**Number of lesions**	
Multiple	106 (92.2)
Solitary	9 (7.8)
**Primary lesions**	**106**
Nodules	58 (50.4)
Papules and plaques	47 (40.9)
Macules/patches	7 (6.1)
Mass/tumor	3 (2.6)
**Secondary changes**	
None	91 (79.1)
Serous and sero-purulent crusts	12 (10.4)
Erosion and ulceration	6 (5.2)
Hemorrhagic crusts and eschar	3 (2.6)
Not indicated	3 (2.6)

**Table 3:** General histologic patterns seen in the study.

General Pattern	*N* (%) *N* = 122
**Infiltrative**	**32 (26.2)**
**Intravascular**	**24 (19.7)**
Infiltrative	6 (25.0)
Nodular	2 (8.3)
Top-heavy	1 (4.2)
**Nodular**	**23 (18.9)**
**Diffuse**	**17 (13.9)**
Infiltrative	4 (23.5)
Nodular	1 (5.9)
Top-heavy	1 (5.9)
**Nodular and infiltrative**	**12 (9.8)**
**Top heavy**	**7 (5.7)**
Nodular	2 (28.6)
Infiltrative	1 (14.3)
**Bottom Heavy**	**6 (4.9)**
Nodular	2 (33.3)
Infiltrative	1 (16.7)
Intravascular	1 (16.7)
**Superficial and deep perivascular**	**1 (0.8)**

General patterns are in bold, other histologic patterns observed within the general patterns are listed.

**Table 4:** Immunohistochemical staining performed in the study.

Patient Code	Primary Site	Immunohistochemistry Studies Performed
18	Nasopharyngeal	AE1/AE3—diffuse, strong, cytoplasmic; CEA—negative
25	Breast	CK7—focal, strong, membranous
39	Melanoma	S100—diffuse, moderate stain, cytoplasmic
61	Breast	CK7—diffuse, strong, cytoplasmic; CK20—negative; CEA—negative
62	Breast	CK7—diffuse, strong, cytoplasmic; CEA—focal scattered staining of cells, cytoplasmic
64	Unknown	CK7—diffuse, strong, cytoplasmic
69	Breast	CK7 strong, cytoplasmic, focal
71	Hematologic	CD20—negative; CD3—negative; MPO—diffuse, strong, cytoplasmic
81	Breast and Lung	CD34—negative, but highlights vascularization; CK7—negative; CEA-negative; CD10—scattered staining; ER—diffuse, strong, nuclear
89	Hematologic	MPO—scattered staining, cytoplasmic; CD3—diffuse, strong, cytoplasmic; CD20—negative; CD10—strong, diffuse, cytoplasmic; CD1a—negative
91	Melanoma	HMB45—diffuse, strong, cytoplasmic
93	Melanoma	HMB45—diffuse, strong, cytoplasmic
96	Hematologic	CD7—scattered staining, weak, cytoplasmic; CD5, diffuse, strong, cytoplasmic margins; CD2—scattered, strong, dot-like pattern; CD3—diffuse strong cytoplasmic
101	Hematologic	Tdt—negative; MPO—strong, scattered, cytoplasmic
105	Breast	CK7—diffuse, strong, cytoplasmic margin; CEA—scattered, strong, cytoplasmic margin
106	Renal	AE1/AE3—strong, cytoplasmic; EMA—strong, cytoplasmic margins; CD10—strong, cytoplasmic

**Table 5:** Differential diagnoses for cutaneous metastasis provided by clinicians.

Clinical Differential Diagnosis Given along with Cutaneous Metastases	
Benign	Malignant
Infection Hansen’s disease, erythema nodosum Erysipelas Septic vasculitis Herpes zoster Bullous impetigo Inflammatory Morphea Radiation dermatitis Lymphedema Tumor Seborrheic keratosis Pyogenic granuloma Lymphangioma	Tumor Angiosarcoma Merkel cell carcinoma Basal cell carcinoma Squamous cell carcinoma Paget’s disease

**Table 6:** Summary table for different retrospective studies on cutaneous metastasis.

	Saeed et al., 2004 + [1]	Nibhoria et al., 2014 * [3]	Sariya et al., 2007 [5]	Fernandez-Flores, 2010 * [8]	El Khoury, 2014 [9]	Kaplan, 2019 + [10]	Hu et al., 2008 [13]	Benmously et al., 2008 [14]	Gan et al., 2015 [15]	Wong et al., 2014 [16]	Chopra et al., 2010 [17]
Region	North America	Asia	North America	Europe	Middle East	South America	Asia	Africa	Asia	North America	Asia
Number of years reviewed	10	9	15	13	18	12	20	14	10	25	14
Patient (*N*)	77	9	51	69	72	96	141	14	35	401	14
Sex											
Males	75	4	21	32	22	38	61	8	14	Not specified	3
Females	2	5	30	37	50	58	80	6	21		11
Mean age (range)	62 (38–83 y.o.)	60 (30–72 y.o)	62 (37–86 y.o.)	(45–90 y.o.)	55.2 (19–81y.o.)	67.95 (28–96 y.o.)	60.8 (22–88 y.o)	63.5 (53–96 y.o)	65 (41–88 y.o)	Not specified	Not mentioned
Top 3 sites	Abdomen/groin	Chest Chest and abdomen Face, scalp and trunk	Upper trunk and abdomen Head and neck (Scalp)	abdomen	Chest Head and neck Back	Abdomen Extremities Thorax	Chest Abdomen Scalp	Thorax Abdomen Head and neck	Chest Pelvis scalp	Not specified	Sternum abdomen
Most common cancer											
Male	Lung, melanoma	Non-Hodgkin’s lymphoma, Renal Cell CA	Lung	Lymphoma	Laryngeal CA	Melanoma	Breast, lung	Lung	Unknown primary	Breast (Sex unspecified)	Gastric, colon, lung
Female	GI, GU Breast	Breast	Breast	Breast	Breast	Breast	Breast, lung, colorectal	Breast	Breast		Breast
Most common morphology	nodules	Plaques, nodules	Solitary nodules	nodules	Solitary nodule	Multiple nodules	Multiple nodules	nodules	Dermal nodules	Not specified	Solitary nodule

*: Included non-Hodgkin’s lymphoma; +: included melanoma in the studies.

## Data Availability

Data contained within the article.
